# Ethanol affects fibroblast behavior differentially at low and high doses: A comprehensive, dose-response evaluation

**DOI:** 10.1016/j.toxrep.2021.05.007

**Published:** 2021-05-18

**Authors:** Neelakshi Kar, Deepak Gupta, Jayesh Bellare

**Affiliations:** a Department of Chemical Engineering, Indian Institute of Technology Bombay, Powai, Mumbai, Maharashtra, 400076, India; b Wadhwani Research Centre for Bioengineering, Indian Institute of Technology Bombay, Powai, Mumbai, Maharashtra, 400076, India

**Keywords:** Ethanol, Fibroblast cells, Hormesis, MTT activity, Mitochondria, Cellular stress-response

## Abstract

This study aims to develop a comprehensive understanding of effects of low and high doses of ethanol on cellular biochemistry and morphology. Here, fibroblast cells are exposed to ethanol of varied concentrations [0.005−10 % (v/v)] to investigate cellular activity, cytoskeletal organization, cellular stiffness, mitochondrial structure, and real-time behavior. Our results indicate a sharp difference in cellular behavior above and below 1 % ethanol concentration. A two-fold increase in MTT activity at low doses is observed, whereas at high doses it decreases. This increased activity at low doses does not involve cell proliferation changes or mitochondrial impairment, as seen at higher doses. Moreover, the study identifies different types of mitochondrial structure impairment at high doses. Morphologically, cells demonstrate a gradual change in cytoskeletal organization and an increase in cell stiffness with increase in doses. Cells exhibit adaptation to sub-toxic doses of ethanol, wherein recovery from ethanol-induced stress is a dose-dependent phenomenon. Cell survival at low doses and toxicity at higher doses are attributed to mild and strong oxidative stress, respectively. Overall, the study provides a comprehensive understanding of dose-dependent effects of ethanol, manifesting its biphasic or hormetic response, biochemically, at low doses and illustrating its toxicological effects at higher doses.

## Highlights

• Ethanol exhibits hormetic response in terms of cellular activity.
• 1 % (v/v) ethanol concentration demarcates non-toxic and toxic range.
• Different types of mitochondrial impairment identified at high dose.
• Cellular toxicity is accompanied by an increase in cellular stiffness.
• Dose-dependent cellular stress response to toxicity is observed.

## Graphical abstract

**Figure:**
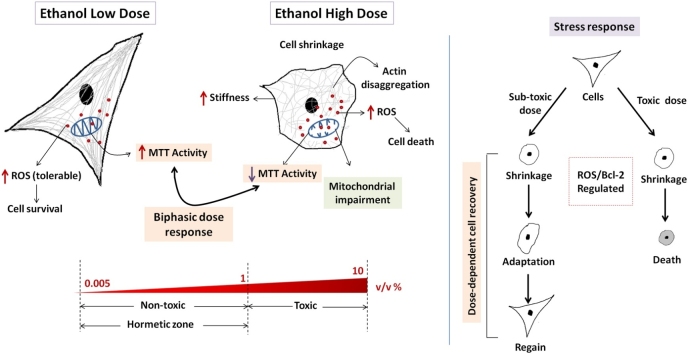

## 1 Introduction

Alcohol is one of the most widely consumed recreational drinks in the modern world. Excessive consumption of alcohol leads to numerous health conditions and is considered a major risk factor for the global burden of diseases [1]. However, there also exist reports of the beneficial effect of alcohol specifically in lowering the risk of coronary heart diseases, diabetes, dementia, and osteoporosis when consumption is limited to moderate to a low level [2]. Moderate alcohol consumption refers to 1 drink/day for women and 2 drinks/day for men, where 1 drink accounts for 14 g of pure alcohol [3]. Thus, alcohol tends to show a dual response, a phenomenon known as Hormesis. Hormesis is defined as “a process in which exposure to a low dose of a chemical agent or environmental factor that is damaging at higher doses, induces an adaptive beneficial effect on the cell or organism” and is characterized by its distinct inverted U shaped or J shaped dose-response curve [4,5]. Though the hormetic effect of ethanol has been studied epidemiologically, cellular studies pertaining to biphasic dose-response to ethanol are limited. Moreover, a recent contradiction on this aspect of ethanol further mandates the need to provide a better toxicological understanding of its dose dependencies [1].

Cellular response to ethanol exposure has been studied with a vast number of cell lines including trophoblasts [6], aortic smooth muscle cells [7], teratocarcinomas [8], lymphocytes, and hepatocytes [9,10]. These studies indicate cytotoxic as well as the antiproliferative effects of ethanol on cells. Though cytotoxicity to ethanol is attributed to the induction of oxidative stress [11], it is both concentrations as well as exposure time-dependent phenomenon [8]. In cellular systems, the hormetic effect of ethanol was studied in nucleoside triphosphate diphosphohydrolases (NTPDase) and 5’-nucleotidase activities of rat platelets. Increased NTPDase activity was observed at an ethanol concentration of 0.8 and 2 g/kg whereas a decrease in Adenosine-5′-triphosphate (ATP) and Adenosine-5′-diphosphate (ADP) hydrolysis was observed in 4, 6, and 8 g/kg of ethanol [12]. Cytoprotective action of ethanol was observed wherein exposure to low concentrations of ethanol (1−2 mM) led to reduced necrosis in hepatocytes [9]. The neuroprotective action of ethanol was also reported at 0.1 % ethanol concentration in hippocampal cells [74] .

However, most of the studies done in literature cover a narrow concentration range, investigating very few parameters, which does not highlight the gradual observable change in different cellular properties. To the best of the authors’ knowledge, this is the first study that strategically and systematically covers a wide range of ethanol treatment doses from trace [0.005 % (v/v) or ∼ 0.085 mM] to a significantly higher amount [10 % (v/v) or ∼1.7 M] of ethanol concentration and investigates its effect on many parameters. This range of ethanol concentration represents both, trace quantities of alcohol which is incidentally present in a number of food, drinks, and medicinal items [13,14], as well as a high dose of alcohol which may intentionally be consumed in the form of alcoholic beverages. Furthermore, this range also covers ethanol concentrations generally considered physiologically relevant in *in vitro* experimental set up i.e. 10−100 mM, with 25 mM ethanol mimicking 0.08 % BAL (Blood Alcohol Level) [15].

This study explains the phenomenon of biphasic or hormetic dose-response of ethanol and evaluates its toxicological profile both at low as well as high doses. It provides an experimental and evidence-based insight into several endpoint effects of ethanol over a wide concentration range. The idea is to understand the nature of the dose-response relationship of ethanol on fibroblast cells by implementing strong experimental design features such as the inclusion of low as well as high doses, adequate number of doses, proper dose spacing, and evaluation of several endpoints. Most of the toxicological assessment studies reported in literature overlook these design conditions, thus missing out the full picture of the complete response pattern [16]. In this study, we hypothesize that stimulatory response to ethanol in cells can be estimated prominently at low doses by introducing a systematic experimental strategy that covers the above-mentioned design criteria. Here, we speculate hormetic response in mouse fibroblast cells upon ethanol exposure for some of the endpoints under consideration viz. cellular activity, reactive oxygen species (ROS) generation, morphological variations in cytoskeletal organization, and cell membrane conformation, and cellular stiffness. Since mitochondrion is known to be one of the major organelles affected by ethanol [17], we anticipate structural defects in mitochondria and test the same. We further attempt to understand that how cells respond to ethanol-induced stress and if adaptive stress response can be traced in a real-time scenario. With alcohol being widely consumed all over the world, such a study can help open new avenues of addiction biology by understanding the threshold of toxicity, tolerance, and adaptability, even at a cellular level.

## 2 Materials and methods

### 2.1 Materials

Chemicals such as Dulbecco’s Modified Eagle’s Medium (DMEM), l-Glutamine, Fetal Bovine Serum (FBS), Trypsin-EDTA solution, Antibiotic-Antimycotic solution (10000 U/mL Penicillin, 10 mg/mL Streptomycin and 25 μg/mL Amphotericin B in 0.9 % normal saline for 100 X), MTT dye, Dimethyl Sulfoxide (DMSO), Trypan blue, Dulbecco’s Phosphate Buffered Saline (DPBS), Tris-EDTA, Propidium iodide (PI), Ribonuclease A (RNase A) and Triton X-100 were purchased from Himedia, India. Molecular probes, Fluorescein isothiocyanate-Phalloidin (FITC-Phalloidin), and 4′,6- diamidino-2-phenylindole (DAPI) were obtained from ThermoFisher Scientific, USA. DCFDA, 3,3′-dihexyloxacarcocyanine iodide (DiOC_6_), Rotenone, 4-(2-hydroxyethyl)-1-piperazineethanesulfonic acid (HEPES) buffer, disodium phosphate (Na_2_HPO_4_) 25 % (v/v) glutaraldehyde, paraformaldehyde, osmium tetroxide, uranyl acetate, and lead citrate were purchased from Sigma Aldrich, USA. The kits used in this study such as the SOD Assay kit and Epoxy Embedding Medium Kit were procured from Sigma Aldrich, USA (Cat. No.:19106 and Cat. No.: 45359-1EA-F respectively), and the ATP Determination kit was purchased from Thermo Scientific, USA (Cat. No.: A22066). The absolute ethanol used in this study is of HPLC grade (Commercial Alcohols, Greenfield Global, Canada). All the reagents and chemicals are of analytical grade.

### 2.2 Maintenance of cell line

Mouse embryonic fibroblast cell line, NIH 3T3 (National Centre for Cell Sciences, Pune, India) was cultured in DMEM media supplemented with 10 % FBS, 1 % l-Glutamine, and 1 % antibiotic-antimycotic solution. Fibroblasts were maintained in their optimal growth conditions of 37 °C, in humidified 5 % CO_2_ incubator (ThermoFisher Scientific, USA).

The next sub-sections describe the experimental methods used for this study. The effect of ethanol on different cellular properties has been investigated over a concentration range of 0.005−10 % (v/v), with 0, 0.005, 0.01, 0.05, 0.1, 0.5, 1, 3, 5, 7 and 10 % (v/v) concentration points, used strategically as per the requirement of experiments.

### 2.3 MTT assay

MTT Assay was done to determine cell viability and activity. Here, cells were seeded in 24 well-plates (0.5 × 10^5^ cells/well) and exposed to 0.005, 0.01, 0.05, 0.1, 0.5, 1, 5 and 10 % (v/v) of ethanol premixed with culture media for 48 h (37 °C, 5 % CO_2_). The final volume of media used in this well-plate format was 500 μL/well, thus the amount of ethanol exposed to the cells corresponds to 0.025, 0.05, 0.25, 0.5, 2.5, 5, 25, and 50 μL, for the above-mentioned concentrations. Also, it should be noted that lower concentrations (0.005 and 0.01 %) were prepared by dilution from 1 % for all the experiments. After 48 h of incubation, 100 μL MTT (5 mg/mL in PBS) was added and again incubated for 4 h at 37 °C. Finally, the absorbance was measured by spectrophotometer (SpectraMax M2 Plate Reader, Molecular Devices, USA) at 570 nm after the addition of 1 mL of DMSO. MTT (tetrazolium salt) is a yellow-colored dye that is converted to purple-colored formazan crystals by metabolically active cells. The insoluble formazan is then dissolved in DMSO. The assay was done according to its standard protocol [18]. Untreated cells were taken as the negative control (referred to as ‘control’ in the entire manuscript). 0.5 % Triton X-100 treatment group was taken as a positive control as it is a detergent known to be toxic to cells, thus inhibiting cell metabolic activity and lowering the absorbance value. For all the experiments, 3 wells per treatment group were taken in each dataset, and at least 3 independent datasets were generated. Moreover, the blank correction was done using samples without cells for all the biochemical experiments, and the corrected values have been presented in the study.

The percentage MTT activity with respect to control was calculated using Eq. (1) given below.

(1) $$\%\;MTT\;Activity=\frac{Absorbance\;of\;treated\;cells}{Absorbance\;of\;control\;}\times 100$$

### 2.4 Determination of cellular ROS level

Intracellular ROS was determined because the metabolism of ethanol involves the production of ROS, which induces oxidative stress [11]. DCFDA assay was used for its detection. DCFDA is a cell-permeable probe that is oxidized to fluorescent compound dichlorofluorescein (DCF) with the help of cellular esterases and ROS produced by cells [19]. In this experiment, cells were grown in 96-black bottom well plates (1 × 10^4^ cells/well) and exposed to different concentrations of ethanol as cited in Section 2.3 ‘MTT Assay’. In 96 well-plate format, the final volume of media used was 100 μL/well, which corresponds to 0.005, 0.01, 0.05, 0.1, 0.5, 1, 5, and 10 μL of ethanol for 0.005, 0.01, 0.05, 0.1, 0.5, 1, 5, and 10 % concentrations. 100 μL of 10 μM DCFDA (prepared in DPBS) was then added, and cells were provided with an optimal temperature of 37 °C for 30 min, for the reaction to take place. Extra DCFDA solution in the wells was discarded and DPBS was added to stop the reaction. The fluorescence intensity was measured in terms of the Relative Fluorescence Unit (RFU) in a spectrophotometer with an excitation/emission wavelength of 485/528 nm. Untreated cells were taken as control. Percentage DCFDA fluorescence with respect to control was calculated using Eq. (2) given below.

(2) $$\%\;DCFDA\;fluorescence=\frac{RFU\;of\;treated\;cells}{RFU\;of\;control}\times 100$$

### 2.5 Cell count assay

Cell counting was done to determine cell viability. Here, cell growth and exposure parameter were kept the same as cited in Section 2.3, ‘MTT Assay’. Live cells were then counted by dilution with 0.4 % Trypan Blue in Haemocytometer. Untreated cells were taken as control. Percentage relative cell proliferation of treated cells with respect to control was calculated using Eq. (3) given below.

(3) $$\%\;Relative\;Cell\;Proliferation=\frac{No.\;of\;treated\;cells}{No.\;of\;cells\;in\;control}\times 100$$

### 2.6 Analysis of apoptosis

Cells were grown on 6-well plates (1 × 10^6^ cells/well) and exposed to the same ethanol concentrations as cited in Section 2.3, ‘MTT Assay’. In 6-well plate format, the final volume of media used was 1 mL/well, which corresponds to 0.05, 0.1, 0.5, 1, 5, 10, 30, and 50 μL of ethanol for 0.005, 0.01, 0.05, 0.1, 0.5, 1, 3, and 5 % concentrations. The cells were then harvested, resuspended in DPBS, and centrifuged (2500 × g, 5 min) to obtain cell pellets. DPBS was then aspirated off and cells were fixed by adding ice-cold 70 % ethanol and stored at 4 °C for 1 h. Cells were then centrifuged at 2500 × g and ethanol was discarded. Cell pellets were resuspended in 0.5 mL PBS and 0.5 mL DNA (Deoxyribonucleic acid) extraction buffer [0.2 M Na_2_HPO_4_ and 0.1 % Triton X-100 (v/v)] and incubated at room temperature for 5 min. The cells were further centrifuged and washed with DPBS and resuspended in DNA staining solution (25 μg/mL PI and 50 μg/mL RNase A). The processing of cells was done by following the standard protocol [20]. Untreated cells were taken as control. Cells were then analyzed for apoptosis by flow cytometry [BD (Becton, Dickson and Company) FACS Aria Special Order System, BD Biosciences, USA] using a 488 nm excitation laser filter.

### 2.7 Estimation of SOD activity

SOD is an antioxidant enzyme that ameliorates the toxic effect of superoxide free radicals and protects the cells from the damaging effect of ROS [21]. SOD activity was measured to check the ROS defense machinery of ethanol-treated cells. Cell growth was kept the same as cited in Section 2.6, ‘Analysis of apoptosis’, and ethanol concentrations were taken as 0.01, 1, 3, and 5 % (v/v) for 48 h. Here, representative concentrations across the whole concentration range were taken. One low and two high doses of ethanol were taken, along with 1 % as an intermediate dose to evaluate the trend. 5 % ethanol concentration was included to follow the trend of the outcome which seemed incomplete otherwise. The same concentrations were also used in the ATP experiment for the same reason cited above. To prepare cell lysate, 30 μL of RIPA lysis buffer was added to the cell pellet obtained and incubated in ice for 15 min, to maintain enzyme stability [22]. The cells were then sonicated for 2 s, three times, with 1 min rest in between (i.e. 2 s each at times cited as 0, 1, and 2 min). This was followed by another round of ice incubation for 15 min. Finally, the cells were centrifuged at 13000 × g at 4 °C for 5 min and the supernatant was analyzed for SOD activity. Untreated cells were taken as control. The kit was used as per the manufacturers’ instructions.

### 2.8 Analysis of mitochondrial membrane potential (MMP)

MMP was analyzed to evaluate mitochondrial health of ethanol-treated cells, as ethanol is known to impair mitochondria [17], and may also result in erroneous interpretation of MTT assay [23]. DiOC_6_, a lipophilic cationic dye, has been used for comparative assessment of MMP as it stains mitochondria owing to its high negative potential [24,25]. Cells grown on 6-well plate were exposed to 0.01, 1 and 3 % (v/v) ethanol for 48 h. Here, one low and one high dose of ethanol was taken, along with 1 % as an intermediate dose to evaluate the trend. These concentration points were also used in other experiments to be discussed in the next sections. Harvested cells were suspended in 10 nM DiOC_6_ dye and incubated at 37 °C for 15 min [24]. Cells were then centrifuged and washed with PBS and DiOC_6_ fluorescence intensity measured in the flow cytometer. Untreated cells were taken as negative control (referred to as control). Cells treated with 5 μM rotenone were taken as a positive control as rotenone is known to depolarize mitochondria [26]. Data analysis was done in FlowJo™ Software V10.0.8. (Becton, Dickinson and Company, U.S.A.).

### 2.9 Ultrastructure of mitochondria

Ultrastructure of mitochondria was observed using TEM, to determine the occurrence of ethanol-induced structural defect of mitochondria at low as well as high doses. Cell growth and exposure parameter were kept the same as cited in Section 2.8, ‘Analysis of mitochondrial membrane potential’. Cells were then subjected to primary fixation with 3 % glutaraldehyde (6 h, 4 °C) followed by post-fixation with 1 % osmium tetroxide (2 h, 4 °C) to preserve the cellular ultrastructure in its near-original form. Washing of sample was done after each step with 0.1 M cacodylate buffer, pH 7.3. Then, successive dehydration was done with 25, 50, 75, 90, and 100 % (v/v) ethanol for 30 min. The samples were then washed with propylene oxide (PO) for 10 min. This was followed by graded treatment of samples with PO/Epon (epoxy resin) 1:1 and 1:3 for 30 min followed by only Epon for 1 h. The samples were then kept in the oven (60 °C, 24 h). Blocks obtained were sectioned using ultramicrotome [Ultramicrotome Leica EM UC7 (Electron Microscope Ultracut 7)] to obtain around 40−70 nm thin sections on 200 mesh copper grids. The grids were then stained with 2 % uranyl acetate and 1 % lead citrate and observed in TEM [HRTEM-JEM-2100 (High-Resolution Transmission Electron Microscope-JEOL Electron Microscope), 200 kV].

### 2.10 Determination of ATP production

As ATP production is the most important function of mitochondria, it was determined to check the functional integrity of mitochondria in ethanol-treated cells. Cells grown on 24-well plate were exposed to 0.01, 1, 3 and 5 % (v/v) ethanol for 48 h. Cells were then lysed (Tris-EDTA buffer of pH 7.4) to release intracellular components and heated at 97 °C for 20 min. Heating was done to inactivate ATP degrading enzymes present in the cell lysate. The samples were then cooled, centrifuged (13000 × g, 10 min), and analyzed with the help of an ATP determination kit. The kit measures the luminescence intensity of the sample. The procedure was followed as per the manufacturers’ instructions. Untreated cells were taken as control. Percentage ATP production with respect to control was calculated using Eq. (4) given below.

(4) $$\%\;ATP\;production=\frac{RLU\;of\;treated\;cells}{RLU\;of\;control}\times 100$$

where RLU is the Relative Luminescence Unit.

### 2.11 Time-lapse live imaging

Time-lapse imaging was done to evaluate the real-time behavior of cells in presence of ethanol. 0.01, 1, and 3 % (v/v) ethanol were added to cells grown in a 24-well plate (0.5 × 10^4^ cells/well) and time-lapse imaging was done in Differential Interference Contrast (DIC) mode (Zeiss Spinning Disc Confocal Microscope, Germany) at 10× magnification for 22 h, at an interval of 15 min. DIC technique of optical microscopy was used to improve the contrast of unstained or otherwise transparent samples.

### 2.12 Fluorescence imaging

Morphology of cell cytoskeleton (actin) was observed through fluorescence imaging. Cells grown on glass coverslip in 24-well plate were exposed to ethanol [0.01, 0.1, 1, 3, 7 and 10 % (v/v)] for 48 h. For analysis of cell morphology, the fine grain concentrations were chosen including 7 % to trace the gradual observable change in morphological structures upon ethanol exposure. The cells were then fixed (4 % paraformaldehyde) and permeabilized (0.2 % Triton X-100, 10 min). This was followed by FITC-Phalloidin staining (4 °C, 4 h) and DAPI staining (room temperature, 10 min) and observed under Zeiss Laser Scanning Confocal Microscope (Zeiss LSM 780 Confocal Microscope, Germany). Multiline-argon laser and multiphoton laser were used for imaging green (FITC, excitation wavelength of 488 nm) and blue (DAPI, excitation wavelength of 750 nm) stain respectively.

### 2.13 Quantification of cytoskeletal anisotropy

The alignment of actin filaments of the ethanol-treated cells was quantified to determine cytoskeletal disorganization. The anisotropicity of the fibrillar structure of actin microfilaments was quantified via FibrilTool, an ImageJ plug-in. Fluorescence images obtained from confocal microscopy were used to calculate the cytoskeletal anisotropy. The standard protocol for using this plug-in in ImageJ software was followed in this study [27], the details of which can be found in the supplementary information, SI 1 of Supplementary Material 1.

### 2.14 Scanning electron microscopy

Cell surface morphology was studied with E-SEM [Environment Scanning Electron Microscopy, FEI (Field Electron and Ion Company), Quanta 200, USA]. Preparation of cells and treatment parameters were the same as reported in Section 2.12, ‘Fluorescence imaging’. Fixation of cells was done with 3.5 % (v/v) glutaraldehyde and subjected to graded ethanol dehydration, as reported in Section 2.9, ‘Ultrastructure of mitochondria’. This was followed by Critical Point Drying (CPD) of coverslips (35 °C, 1 bar in Leica EM CPD300, Germany), a process to remove liquid from cells without affecting their microstructural integrity [28]. Before imaging, the coverslips were coated with platinum for 300 s with a sputter coater (JEOL JFC-1600, Japan).

### 2.15 Atomic force microscopy

Atomic Force Microscopy was used to measure the cellular stiffness. Preparation of cells and ethanol treatment parameters were kept the same as reported in Section 2.12, ‘Fluorescence imaging’, since the stiffness of cells is associated with cell cytoskeleton. Coverslips were then placed on a 60 mm dish and media were changed to the one with an extra supplement of 20 mM HEPES. Cells were then tapped with a silicon nitride, 10 kHz probe. Force curves were obtained with AFM (MFP-3D-BIO Inverted Optical AFM - Asylum Research) and fitted in the Hertz Model of elasticity in Asylum Research software using Igor Pro 6.37 (WaveMetrics, Lake Oswego, OR, USA) to obtain its Young’s Modulus value (a measure of stiffness i.e. higher the Young’s Modulus, higher is the stiffness).

### 2.16 Statistical analysis

The statistical significance analysis was done by One Way ANOVA (Analysis of Variance) followed by post-hoc Tukey’s test in OriginPro 9.1 software [29]. Data from all the experiments have been represented as mean ± standard error of the mean (SE), and p-values less than 0.05, 0.01, and 0.005 were considered statistically significant. Data obtained from three independent experiments (n = 3) and done in triplicates are reported in this study.

## 3 Results

### 3.1 MTT activity, cell proliferation, and apoptosis

**Fig. 1:**
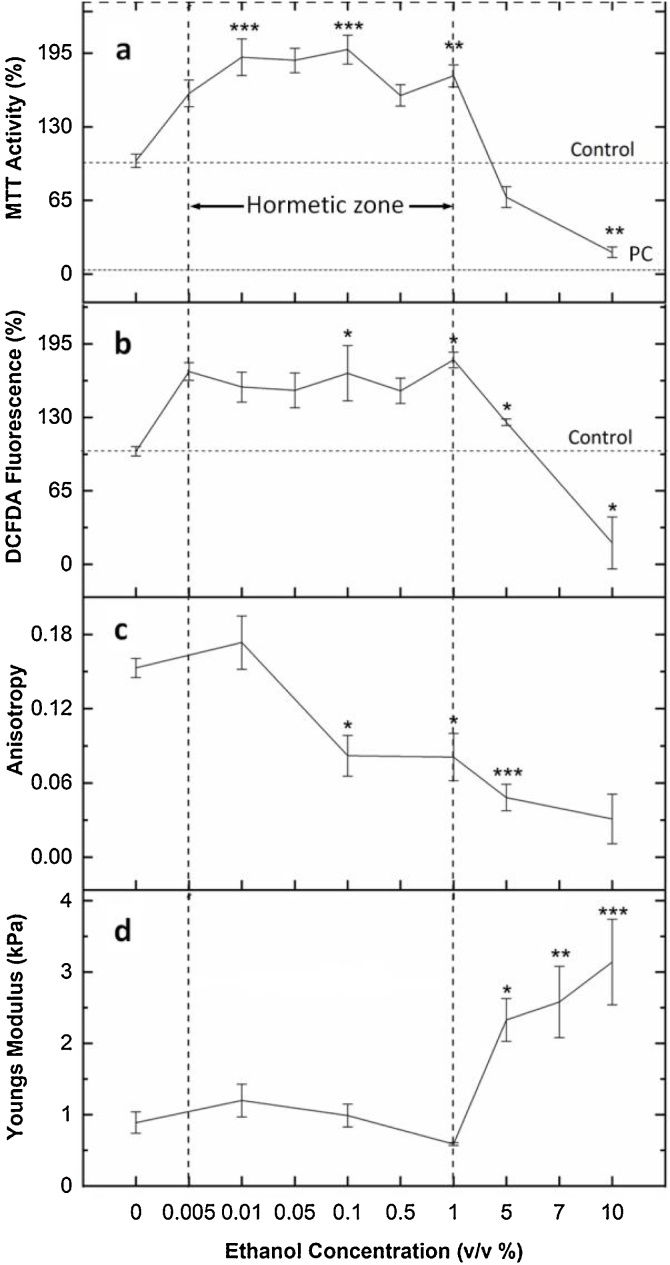
(**top to bottom**) a) MTT Cell Viability Assay. MTT activity is highest in the range of 0.005-1 % (v/v) of ethanol treatment which signifies the stimulatory region. Above 1 %, cell viability decreases. PC: Positive Control. b) ROS activity of fibroblast cells. An increase in ROS production can be seen in the concentration range of 0.005-1 % (v/v). c) Quantification of actin microfilament anisotropy as calculated from the Fibril Tool plugin of ImageJ. An increase in ethanol concentration leads to cytoskeletal disorganization (0.1 % and above). d) Fibroblast cell stiffness. Cells are soft in the 0.01-1 % (v/v) range and become stiff above 1 % ethanol exposure. Data are represented as Mean ± SE (n = 3). *P < 0.05, **P < 0.01, ***P < 0.001 with respect to control.

**Fig. 2:**
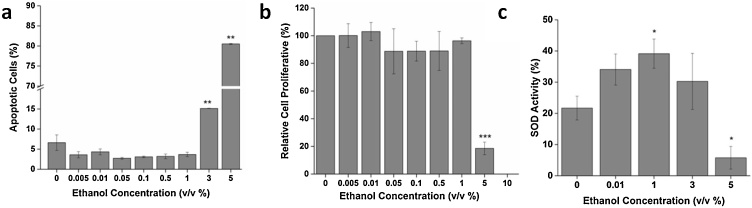
a) Analysis of apoptotic cells depicts the occurrence of apoptosis above 1 % (v/v) ethanol concentration. b) Cell count assay confirms that there is no ethanol-induced cell proliferation at low doses as compared to control. c) SOD activity assay suggests that SOD activity increases with an increase in ethanol concentration up to 1 %, and then the activity is inhibited. Data are represented as Mean ± SE (n = 3). *P < 0.05, **P < 0.01, ***P < 0.001 with respect to control.

Fig. 1a illustrates that the MTT activity of ethanol-treated cells is almost double the activity of control in the concentration range of 0.005–1 % (v/v). Above 1 %, a sharp decrease in activity is observed. To check whether the increase in MTT activity at the low doses, was due to increased cellular proliferation or because of increased cellular activity, a cell count assay was done. As seen in Fig. 2b, there is no ethanol-induced cellular proliferation found in the concentration range of 0.005–1 % which indicates that the increase in MTT activity was due to the increase in cellular activity. However, above 1 %, toxicity ensues which can be seen in the form of decreased cell count as well as a steep drop in the activity curve. Analysis of apoptotic cells upon PI staining further confirms that exposure to ethanol above 1 % results in loss of cell viability as seen in Fig. 2a.

Thus, the inverted U-shaped curve of MTT activity signifies biphasic dose-response or hormetic response of cells to ethanol, which signifies increased ability of cells to metabolize tetrazolium salts at a low dose. Here, the maximal hormetic stimulatory response (MHSR) was found to be ∼198 % at 0.1 % ethanol concentration. This identification of true “max” in the hormetic curve was possible due to the incorporation of a higher number of concentration points in the stimulatory range. The study thus substantiates the importance of a strong study design involving ≥ 6 doses when dealing with low dose effects [30]. Interestingly, the hormetic phenomenon is not only observed in fibroblast cells but also other types of cell lines such as Human Embryonic Kidney Cells (HEK 293) and Hepatocarcinoma cells (HepG2), presented as Fig. S2 of Supplementary Material 2.

Furthermore, it should be noted that covering a large dynamic range of the concentration interval [0.005−10 % (v/v)] necessitates the selection of points (concentrations) at wide intervals and some degree of coarse-graining. Thus, the representative concentrations covering the entire range have been taken strategically in this study. Amongst these coarse grain concentrations, we could establish the trend for biphasic response and found that the transition of behavior occurs at around 1 %; though the exact point of transition would have been better known with the inclusion of finer grain concentration points. Further detailed information on the overall strategy behind the selection of ethanol concentrations for each experiment can be found as supplementary information, SI 2 in Supplementary Material 1.

### 3.2 Estimation of reactive oxygen species

The DCFDA fluorescence provides an estimation of ROS produced by the cells. In Fig. 1b, it is observed that ROS production is higher in the ethanol concentration range of 0.005−1 % (v/v) than that of control. However, the increased ROS production up to a certain limit is not deleterious to cells and is termed as a tolerable level. This study indicates that the tolerable limit for ROS in fibroblast cells is 1.8 times more than that of control, and the tolerable range of ethanol concentration dose is 0.005−1 % (v/v). Within tolerable limit, the amount of ROS is considered to be mild and literature suggests the beneficial role of mild ROS including eliciting cell survival pathways [31,32]. Subsequently, at 5 % and 10 % ethanol treatment, the increase in ROS production is greater than the tolerable limit, thus compromising cell viability and resulting in decreased net DCFDA fluorescence. Thus, the overproduction of ROS induces oxidative stress which is detrimental to cellular health.

### 3.3 Analysis of SOD activity

The SOD activity of ethanol-treated cells increases with ethanol dose up to 1 % (v/v) (Fig. 2c), thereafter it starts decreasing. An increase in SOD activity indicates that cells cope up with ROS produced by ethanol at low concentrations. However, as the ethanol dose increases above 1 %, the oxidative stress increases and SOD activity gets inhibited. The induction of oxidative stress by ethanol is associated with its cellular metabolic pathways which involve the production of oxidative species (ROS) [11]. Initially, when ethanol concentration is below 1 %, ROS production is above physiological level, but antioxidant enzyme activity increases, thus activating cells’ defense machinery against ROS and eliminating its toxic effects. However, an increase in ethanol dose above 1 %, results in increased ROS production, thus inducing oxidative stress. This inhibits antioxidant enzyme activities of the cell, causing cellular damage.

### 3.4 Analysis of MMP and mitochondrial swelling

To evaluate mitochondrial dysfunction due to ethanol exposure, the mitochondrial membrane potential of ethanol-treated cells was studied, along with their light scattering pattern to understand mitochondrial swelling.

**Fig. 3:**
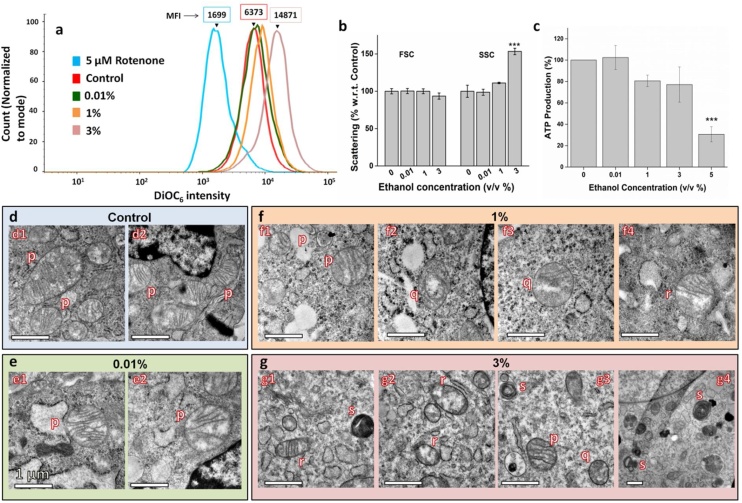
Effect of ethanol on mitochondria a) Fluorescence intensity curve of cells treated with different concentrations of ethanol to analyze Mitochondrial Membrane Potential (MMP). MFI is Median Fluorescence Intensity, which increases with an increase in ethanol concentration. Color coding for the curves are as follows: Red, Control; green, 0.01 % (v/v); orange, 1 % (v/v); magenta, 3 % (v/v); and blue, 5 μM Rotenone. b) Light scattering pattern. FSC/SSC ratio is an index of measurement of mitochondrial swelling. An increase in ethanol concentration results in swelling of mitochondria. c) ATP production. ATP production decreases with an increase in ethanol concentration. d, e, f, and g) Ultrastructure of mitochondria of cells treated with 0 % (Control), 0.01 %, 1 % and 3 % (v/v) ethanol respectively. Notations “p”, “q”, “r”, and “s” denote four types of morphology displayed by mitochondria upon ethanol treatment. “p” represents healthy mitochondria, “q” represents mitochondria with disrupted Inner Mitochondrial Membrane, “r” represents mitochondria with disrupted Outer Mitochondrial Membrane, and “s” represents “onion-like concentric ring” structured mitochondria. Mitochondrial defects can be seen in 1 % and 3 % treatment groups. Data are represented as Mean ± SE (n = 3). Scale bar: 1 μm. ***P < 0.001 with respect to control.

Fig. 3a depicts the DiOC_6_ fluorescence intensity of ethanol-treated cells. Here, the x-axis represents DiOC_6_ fluorescence intensity, whereas the y-axis represents cell count. The position of the peak on the x-axis represents the DiOC_6_ intensity. As seen in Fig. 3a, the fluorescence intensity for 0.01 % (v/v) ethanol-treated cells is almost similar to that of control, as both the peaks almost overlap with each other. However, as the ethanol concentration increases to 1 and 3 %, there is a peak shift towards the right, showing an increase in intensity. The fold increase in MFI (Median Fluorescence Intensity) with respect to control is 1.09, 1.3, and 2.3 for 0.01, 1, and 3 %, respectively. This implies that ethanol at a low dose does not affect the mitochondrial health but with an increase in ethanol concentration, the MMP increases, indicating hyperpolarization of the mitochondrial matrix. 5 μM Rotenone-treated cells, which were taken as the positive control, show a significant decrease in MMP. Rotenone has been used as a positive control since it is known to depolarize mitochondria [26].

Light scattering pattern as analyzed by FACS shows that side scattering increases (SSC) with an increase in the dose (3 %), without any change in forward scattering (FSC) (Fig. 3b). As a result, the FSC/SSC ratio decreases. This light scattering pattern combined with the trend observed with MMP indicates an increase in the volumetric proportion of mitochondria, suggesting mitochondrial swelling [33].

### 3.5 Determination of ATP production

As seen in Fig. 3c, the level of ATP produced at 0.01 % (v/v) is 102 ± 11 % of control which is statistically insignificant as compared to the control. However, the graph shows a decreasing trend from 1 to 5 % ethanol treatment. The values decrease to 80.64 ± 5.37 %, 77.17 ± 16.48 %, and 30.64 ± 7.04 % for 1, 3, and 5 %, respectively. The results indicate that though mitochondrial functional integrity is unaffected at a low dose of ethanol, increasing the dose (1 % and above) adversely affects its function.

### 3.6 Ultrastructure of mitochondria

The ultrastructure of ethanol-treated cells was observed through TEM to specifically analyze the mitochondrial defects. Four major types of morphologies are identified upon ethanol treatment. In Fig. 3d–g these four morphologies have been denoted as “p”, “q”, “r”, and “s”, representing healthy mitochondria, mitochondria with disrupted Inner Mitochondrial Membrane (IMM), mitochondria with disrupted Outer Mitochondrial Membrane (OMM) and “onion-like concentric ring” structured mitochondria, respectively. As seen in Fig. 3e, the mitochondrial structure seems to be unaffected at low dose ethanol treatment of 0.01 % (v/v). The OMM is distinct and IMM forms cristae that are visually optimal. However, at 1_% ethanol and above, deformity arises. At 1_%, some of the mitochondria show disruption of cristae (Fig. 3f2, f3) and others, rupture of OMM (Fig. 3f4). The formation of compartments inside the mitochondria can also be seen (Fig. 3f2). At 3_% ethanol concentration, deformation is more evident. Apart from deformation in OMM and IMM, cristae forming concentric circles within the OMM (Fig. 3g1, g3, and g4) are also observed. Thus, different types of structural damage to mitochondria are observed, with the effect of damage being more prominent with increasing ethanol dose.

### 3.7 Time-lapse study of ethanol-treated cells

**Fig. 4:**
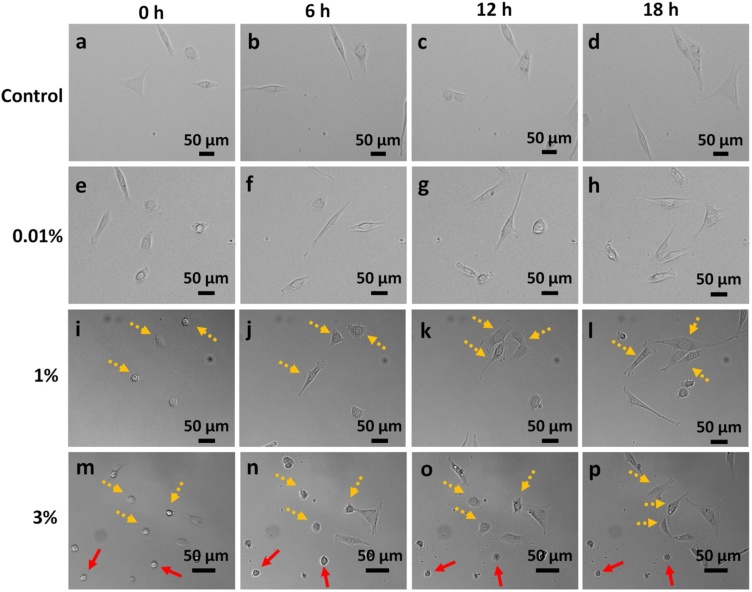
Time-lapse imaging of ethanol-treated cells (a-d) Control; (e-h) 0.01 %; (i-l) 1 %; (m-p) 3 % (v/v) ethanol-treated cells. 0.01 % ethanol-treated cells do not show a significant change in morphology. At 1 % and 3 % ethanol exposure, cells initially shrink, and thereafter, some of them regain their morphology (yellow-dotted arrows), while others die (red arrows). Cell death in 3 % is more than 1 %. Images taken at 10x objective, NA: 0.45.

To investigate the fibroblast behavior immediately after ethanol exposure, time-lapse imaging was done. The magnification of imaging was kept at 10×, so the micrographs presented in the text are of the ‘cropped up’ region of interest. However, the concerned videos have been attached in Supplementary Material 4 (Control), 5 (0.01 % treatment), 6 (1 % treatment) and 7 (3 % treatment). Three different concentrations of ethanol, 0.01, 1, and 3 % (v/v), have been used in this study. As seen in the DIC images of cells in Fig. 4, treatment of cells with 0.01 % ethanol does not cause any change in morphology as compared to control. However, at 1 % and 3 % ethanol concentrations, cells show a propensity for adaptation. 1 % and 3 % ethanol treatment lead to shrinkage of cells within a few minutes of treatment. The shrunken and rounded cells have been indicated by yellow-dotted and red arrows in Fig. 4. Cells denoted by the yellow-dotted arrows are the ones that are dynamic in nature and eventually spread their surface to regain healthy morphology. Whereas, cells denoted by the red arrows are the ones that become static over a period and eventually die. The quantification of these two types of cell fate has been done here.

**Fig. 5:**
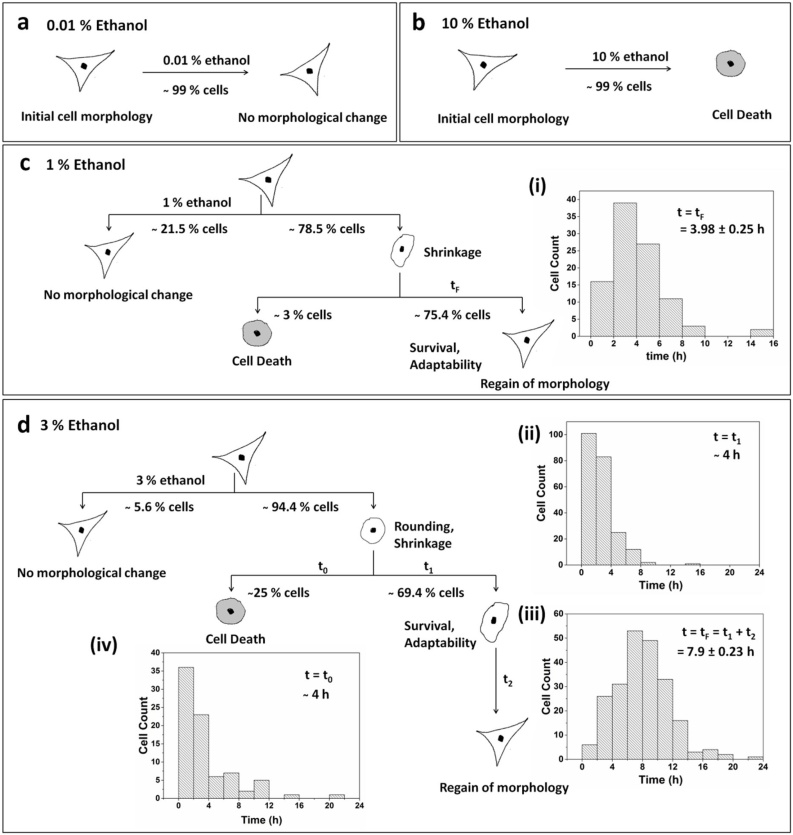
Schematic representation of the fate of cells treated with ethanol. a) 0.01 % (v/v) ethanol treatment does not show any morphological change; b) 10 % (v/v) ethanol treatment leads to cell death; c) 1 % (v/v) and d) 3 % (v/v) ethanol treatment results in cell survival and adaptability. The histogram frequency distribution graph depicts the time taken by cells to i) completely regain original morphology upon 1 % exposure (t_F_ = 3.98 ± 0.25 h). For 3 % exposure, the graphs depict the time taken by cells; ii) to initiate regain in morphology (t_1_ = ~ 4 h); iii) for complete regain in original morphology (t_F_ = 7.9 ± 0.24 h). t_F_ = t_1_+t_2_, where t_2_ is the time taken by cells to regain their morphology from the point of initiation; and iv) to commit to apoptosis or cell death (t_0_ = ~ 4 h).

A population study of about 130 and 305 cells was done for 1 % and 3 % ethanol treatment respectively, and the histograms for frequency distribution were plotted (Fig. 5). Only single cells have been considered for generating the pertaining data. The fate of cells is represented schematically in Fig. 5. When cells are exposed to 1 % ethanol concentration, about 78.5 % of cells shrink and 21.5 % of cells do not show any change in morphology. Amongst the shrunken cells, around 75.5 % of cells initiate recovery immediately and regain their original morphology in 3.98 ± 0.24 h (Fig. 5c i), and only 3 % of cells die. Furthermore, exposure to 3 % ethanol concentration leads to shrinkage and rounding up of around 94.5 % of cells. However, unlike 1 % exposure, cells retain their rounded morphology for some time before initiating its change in shape. The graphs in Fig. 5d ii and d iii indicate that most of the cells (69.4 %) initiate recovery within the first four hours of cell treatment and regain their full morphology in 6–10 h. On average, the stressed cells regain their original morphology in 7.9 ± 0.23 h (Fig. 5d iii). However, a fraction of cells (25 %), which cannot withstand the stress induced by ethanol, undergo cell death. Maximum cell death is observed within the first four hours of treatment (Fig. 5d iv). The result indicates that the period of the initial four hours of ethanol treatment is a significant deciding factor, wherein cells initiate signaling pathways either for survival or death [34]. Also, a small fraction of cells (5.6 %) does not show any change in morphology even after ethanol treatment. Thus, the results of this study suggest that the adaptability of cells to ethanol exposure is a dose-dependent phenomenon. Higher the toxicity more is the time taken by cells to adapt for survival. This can also be interpreted in terms of hormetic adaptive response in temporal context, where low and sub-toxic doses of ethanol prepare the cells in the initial 4−8 h of exposure and acclimatize them for the persistent ethanol-induced stress for 22 h [35,36].

### 3.8 Actin cytoskeletal organization

**Fig. 6:**
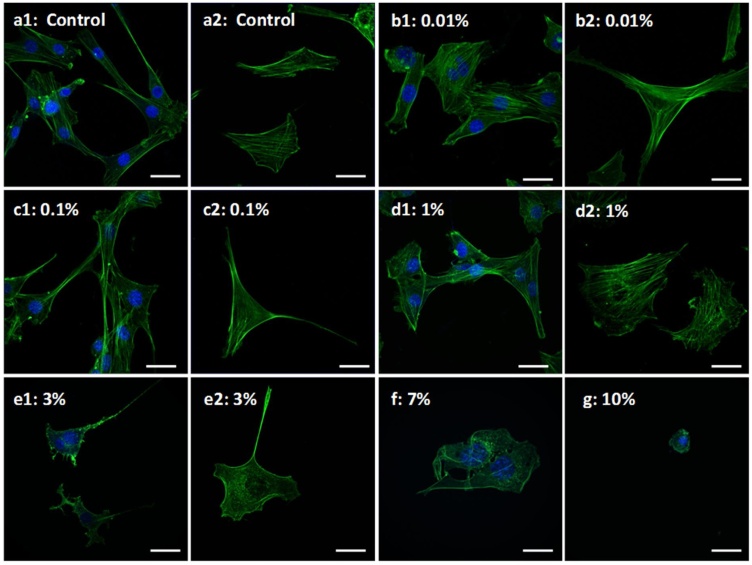
Confocal Micrographs of ethanol treated cells. Figure depicts cellular morphology upon exposure to different concentration of ethanol. a1, a2: Control; b1, b2: 0.01 % (v/v); c1, c2: 0.1 % (v/v); d1, d2: 1 % (v/v); e1, e2: 3 % (v/v); f: 7 % (v/v); g: 10 % (v/v) ethanol. Increase in ethanol concentration leads to actin disorganization and membrane instability. Loss of actin can be seen in e1, f and g. Green: FITC-Phalloidin staining actin filaments; Blue: DAPI staining nucleus; images taken at 63 × (oil) objective, NA: 1.40; Scale bar: 20 μm.

Fig. 6 represents the confocal microscopy images of ethanol-treated and untreated fibroblast cells. As the concentration increases from 0.01 % to 10 % (v/v), a disordering effect on the cell membrane is observed. This is accompanied by shrinkage of cells which ultimately results in cell rounding. In the control group, the actin filaments display a sharp organized morphology striating from one end of the cell to another. However, with an increase in ethanol concentration, their orientation becomes random, leading to their disorganized arrangement. This effect is seen to be prominent in cells exposed to 1 % ethanol and above. Actin disorganization at 1 % and 3 % could not affect cell viability to a greater extent; however, ethanol exposure exceeding 5 % concentration causes severe damage to the cellular structure leading to cell death.

### 3.9 Quantification of cytoskeletal anisotropy

**Fig. 7:**
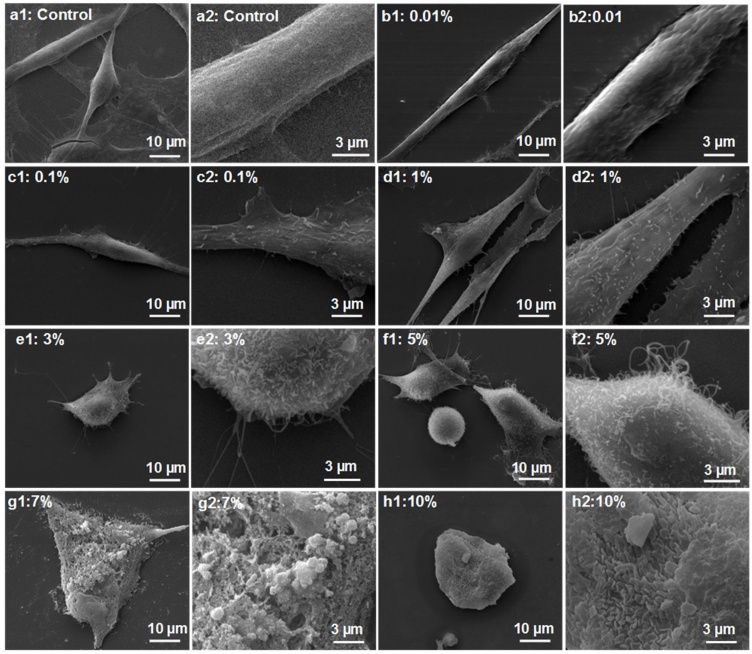
SEM Micrographs of ethanol treated cells. Figure depicts cellular morphology upon exposure to different concentration of ethanol. a1, a2: Control; b1, b2: 0.01 % (v/v); c1, c2: 0.1 % (v/v); d1, d2: 1 % (v/v); e1, e2: 3 % (v/v); f1, f2: 5 % (v/v); g1, g2:7 % (v/v) and h1, h2: 10 % (v/v). a2, b2, c2, d2, e2, f2, g2 and h2 are cropped zoomed out images of corresponding original images. Increase in ethanol concentration leads to membrane instability as well as formation of microspikes and membrane ruffles. Images taken at 5000× magnification.

Fig. 1c represents the anisotropy value of actin filaments against ethanol dose. The graph illustrates that as the ethanol concentration increases; the anisotropy of actin filaments also increases. Within the hormetic range of ethanol treatment i.e. 0.01–1 % (v/v), the treatment initially leads to actin remodeling within the cells with gradual cytoskeletal disorganization upon increasing the treatment concentration. This is well observed with a steep dip in the curve from 0.01 to 1 % of ethanol treatment. Further increase in dose leads to cytoskeletal disaggregation accompanied by even loss of actin filaments (above 1 %) as evident from the fluorescence image Fig. 6e1, f, and g and SEM image in Fig. 7e1, f1, g1 and h1 (discussed in next Section 3.10, ‘Cell surface morphology’).

### 3.10 Cell surface morphology

Fig. 7 represents the ethanol effect on the surface morphology of fibroblast cells from SEM micrographs. As concentration increases from 0.01 to 1 % (v/v), the cell surface becomes coarse with the extrusion of numerous ciliary projections from the cell surface. This roughness might be because of cytoskeletal rearrangement which leads to protrusion of its elements through the cell membrane. This appears in the form of microspikes or filopodial extensions from the cell surface. The appearance of membrane ruffles at 0.1 % ethanol is observed. Above 1 % ethanol exposure, cell shrinking begins with an increase in the number of ciliary projections. At 5 %, rupture of cell membrane and emergence of actin filaments out of the cell is observed. Membrane blebs that appear over the cell surface at 7 % ethanol treatment signify cell death.

### 3.11 Cell stiffness

Cell stiffness measured by AFM is depicted in Fig. 1d. The graph illustrates that with an increase in ethanol concentration from 0.01 to 1 % (v/v), the Young’s Modulus shows a decreasing trend, reducing from 1.2 ± 0.23 kPa to 0.59 ± 0.02 kPa, respectively. This decrease in cell stiffness is attributed to actin disorganization, as the actin cytoskeleton provides mechanical strength and stability to cells [37]. Moreover, ethanol also enhances membrane fluidity, which might further contribute to lowered Young’s Modulus [38]. Furthermore, an increase in ethanol concentration above 1 % increases Young’s Modulus to 2.33 ± 0.3 kPa at 5 % and 3.14 ± 0.6 kPa at 10 %. This increase in cell stiffness is associated with cell shrinkage or reduced cell volume, which leads to crowding of intracellular space with organelles and macromolecules, thus enhancing resistance to strain [39].

Moreover, mechanophysical properties of cells such as cellular stiffness help in understanding the pathophysiology of diseases and disease progression as in the case of malaria (stiffening of diseased erythrocyte upon parasite invasion) and cancer (cell softening promoting metastasis) [40]. Thus, an understanding can be established from this experiment that a toxic dose of ethanol increases fibroblast stiffness.

## 4 Discussion

This study covers certain significant aspects of biochemical, morphological, and biophysical changes occurring in cells upon ethanol treatment with an emphasis on low dose effects. Here, the results show the contrasting behavior of cells exposed to ethanol at low and high concentrations as reflected in the results presented above and to be discussed next. The summary of the overall findings of the study has been presented in Table 1.

**Table 1:** Summary of overall findings of the study.

S.N.	Parameters Tested	Effects on fibroblast cells	
		Non-cytotoxic Range 0.005−1 % (v/v)	Cytotoxic Range > 1 % (v/v)
1	MTT Activity	High	Low
2	Cell viability and proliferation	Viable cells, no effect on proliferation	Cells lose viability
3	ROS generation	High, tolerable at 1 %	High, non-tolerable
4	Mitochondrial Membrane Potential	No change, but slightly high at 1 %	Hyperpolarization at 3 %
5	Mitochondrial volumetric proportion	No change	Increases
6	ATP Production	No change, but statistically insignificant decrease at 1 %	Decreases
7	Mitochondrial structure	No change at 0.01 %; deformity starts at 1 % (compartmentalization, rupture of OMM and IMM observed along with healthy mitochondria)	Mitochondrial defects observed (“onion-like” concentric rings of cristae, rupture of OMM and IMM observed)
8	Real-time cell behavior	No change at 0.01 %; adaptability to survival at 1 %	Adaptability to survival and death at 3 %
9	Cell Stiffness	Low	High
10	Cell Morphology	Actin reorganization, appearance of microfilaments, membrane ruffles	Cell rounding, shrinkage, appearance of membrane ruffles and blebs, protrusion of microfilaments from the cell membrane, cell membrane instability

This study identifies the low-dose stimulation and the high-dose inhibition effect of ethanol in terms of increased cellular activity. Cellular activity here is measured in terms of MTT activity, which signifies the ability of cells to metabolize tetrazolium salts by nicotinamide adenine dinucleotide phosphate (NADPH)-dependent cellular oxidoreductase enzymes of mitochondria [41]. The signal strength of the MTT assay is influenced both by cellular viability and metabolic activity of cells [18]. This study shows that the MTT activity of cells increases at low doses of ethanol (< 1 %), without an increase in the cell count, whereas at high doses (> 1 %) cellular viability is compromised and net cellular activity decreases. The MTT activity graph shows an inverted U-shaped curve. The characteristics of the obtained curve are consistent with that of the hormetic dose-response curve i.e. the extent of stimulation is within the specified upper limit of 2 fold increase from control (here, ∼1.98 fold increase in MTT activity), and width of stimulation falls in 20- < and ≤ 1000- fold range [here, ∼200 fold for 0.005−1 % (v/v)] [42]. Thus, this curve is analogous to the classical hormetic dose-response curve [42]. Moreover, this study covers a wide concentration range, with the incorporation of 6 doses in the stimulatory range of the hormetic curve, thus enabling it to identify the true response optima or MHSR. This experimental design strategy is in line with the fact that an increase in the number of doses below zero equivalent point, increases the amplitude of response optima [30].

Apart from ethanol, numerous other compounds reportedly elicit a hormetic response such as opioids, adrenergic agents, toxic inorganic agents (arsenic, cadmium, mercury, etc.), chemotherapeutic agents (antibacterial, antiviral, antitumor, and antiangiogenesis agents), and herbal extracts (green tea, arnica flower, aloe plant, coriander fruit/berry/seeds, etc.) [2,[42], [43], [44]]. Moreover, MTT has been widely used as an indicator of the hormetic effect of compounds such as anticancer drugs and heavy metals [36,[45], [46], [47], [48]].

As far as ROS is concerned, ethanol at low concentration triggers the production of the above physiological but mild ROS, which in turn elicits the well-known redox signaling pathways needed for cell survival [31,49,50]. However, at higher ethanol concentration, production of ROS exceeds the tolerance level, and induces oxidative stress, thereby affecting the cells adversely by mediating signaling pathways leading to cell death [11]. Our study indicates that the tolerable limit for ROS in fibroblast cells is 1.8 fold greater than control, with 1 % being the upper limit of tolerable ethanol dose. Moreover, a low dose of ethanol also increases antioxidant enzyme activity, as observed with an increase in SOD activity in this study, which in turn ameliorates the ROS toxicity. This is in line with the observation of 0.1 % ethanol inducing enhanced antioxidant activity in hippocampal HT22 cells, thus protecting against glutamate neurotoxicity [74] .

The adverse effects of high doses of ethanol on mitochondria reported in our study which includes mitochondria hyperpolarization and swelling are consistent with that of myocardial cells showing similar ethanol effects [33]. Also, the decrease in ATP production at high ethanol dose which is also observed in hepatic and myocardial cells is attributed to the repressed phosphorylation complexes activities, resulting in overall malfunctioning of the oxidative phosphorylation system [51].

Additionally, this study identifies different types of structural defects of mitochondria at 1 % and above ethanol concentration. Apart from the disruption in IMM and OMM, compartmentalization and concentric onion-like rings of IMM are similar to what has been observed in the literature [52]. Damage to the mitochondrial structure is attributed to increased oxidative stress. Ethanol treatment enhances cellular oxidative stress which mediates the induction of mitochondrial permeability transition (MPT) [17]. MPT is a phenomenon observed in apoptotic or pro-apoptotic cells which are characterized by IMM permeabilization and matrix swelling, ultimately leading to rupture of cristae and OMM [53]. The other two morphologies, compartmentalization and ‘onion ring’ like cristae, are new observations pertaining to ethanol-induced mitochondrial damage. However, both types of morphologies have been observed in the case of mitochondrial myopathy [54]. Compartmentalization occurs in the event of incomplete mitochondrial fusion, where OMM fuses but IMM fails to fuse completely and forms a compartment [54]. Mitochondrial fusion otherwise helps in maintaining functional mitochondria upon induction of stress [55]. Concentric ‘onion ring’ like cristae has also been observed in HeLa (cervical cancer) cells and yeast with downregulated mitochondrial contact site and cristae organizing system [54,56], and in yeast with ATP synthase dimerization defect, resulting from inactivation of ‘e’ and/or ‘g’ subunit of ATP synthase [52]. ATP synthase dimers define cristae structure and form rows along the IMM influencing membrane curvature [52,57].

The current study thus highlights two major outcomes. Firstly, mitochondrial health is not affected by exposure to a very low dose of ethanol, thus supporting the fact that increased cellular activity, as indicated by MTT Assay, is independent of mitochondrial impairment. This firmly confirms the biphasic dose-response of ethanol on cellular activity. Secondly, with an increase in ethanol concentration, MMP and mitochondrial volume increases, which in turn might be the result of a) closure of permeability transition pore signifying induction of apoptosis, described as the VDAC closure model [58,59] or b) increase in anti-apoptotic Bcl-2 protein that enhances the signal of mitochondrial potential sensitive dyes and confers protective action to apoptosis [33,60,61]. We thus hypothesize that at 1 % ethanol exposure, where apoptosis is low with a slight increase in MMP, there is an increase in Bcl-2 protein. However, at 3 % ethanol exposure, where apoptosis is around 15 %, an increase in MMP signifies a tight regulation of apoptosis by Bcl-2 family proteins, where some cells commit to apoptosis owing to stress inflicted by ethanol; and others recover the shock by higher production of anti-apoptotic proteins such as Bcl-2 and heat shock proteins [33,62]. This proposition is based on the fact that cellular apoptosis is regulated by the ratio of pro-apoptotic (Bax, Bak, BH3-only proteins) and anti-apoptotic proteins (Bcl-2, Bcl-X_L_, and Mcl-1) of the Bcl-2 family of proteins [62]. Moreover, the regulation of apoptosis by alteration of the Bax/Bcl-2 ratio has been previously reported in ethanol exposed L02 cells (Normal human liver cells) [63]. The role of anti-apoptotic Bcl-2 protein has also been identified in the cellular adaptation of CHO (Chinese Hamster Ovary) cell line to protein-free culture conditions [64].

Time-lapse imaging of cells suggests that 3 % treatment takes almost twice the recovery time (7.9 ± 0.23 h) than cells treated with 1 % ethanol (3.98 ± 0.24 h), with a lag in the initiation of recovery. Also, the 1 % ethanol treatment group has a greater number of cells which does not show shape change and fewer cells that die. This indicates that cellular stress response to ethanol toxicity is a dose-dependent phenomenon i.e. recovery from mild stress is faster than strong stress. This observation is in line with the fact that cellular stress responses can be of two types depending on the extent of cellular insult. Cells can either adapt to environmental or intracellular stress or respond by killing themselves. The first instinct of cells is to initiate survival pathways, however, if the stress is persistent cells trigger pathways for cell death [34]. The survival of ethanol-treated cells at sub-toxic doses further affirms the theory of the production of anti-apoptotic Bcl-2 proteins [65] as discussed earlier. This also reflects the ability of cells to optimize their existing cellular machinery, biochemical, or genetic, to acclimatize to a new environmental niche [66]. The fraction of cells that do not show any change in morphology upon ethanol exposure indicates the physiological and genetic robustness that might exist in the form of latent genetic variant over a generation of evolution and is expressed upon induction of stress [66].

Our study on fibroblast morphology focuses on the alteration of actin cytoskeletal dynamics as well as cell membrane conformation. Cytoskeletal disaggregation and membrane instability have been observed with an increase in ethanol concentration. Disorganization of actin by ethanol has been previously reported in neural crest cells [67], glial cells [68], and rat pancreatic acinar cells [69]. This is mediated by ROS through Rho signaling pathways [[68], [69], [70]]. Furthermore, ethanol due to its amphiphilic nature, easily diffuses through the phospholipid bilayer, thus imparting a disordering effect on the cell membrane, increasing its fluidity and decreasing the bilayer thickness [38,71]. However, as the ethanol toxicity increases (≥ 5 %), severe morphological damage is observed with a reduction in cell volume, loss of actin filament, and the appearance of blebs. Moreover, an increase in cell stiffness is observed with an increase in toxicity which results from intracellular space overcrowding due to reduction in cell volume and loss of actin at high doses [39].

Overall, this study explores the dual nature of ethanol in terms of fibroblast behavior at low as well as high doses along with its toxicological effects. We have shown here the importance of ethanol concentration at around 1 %, which demarcates a boundary in the entire concentration range of 0.005−10 % into low or non-toxic doses (<1 %) and, high or toxic doses (>1 %). Several parameters have been tested in this study to illustrate the endpoint specificity of dose effects. Amongst all the endpoints evaluated, the hormetic effect is observed in terms of cellular activity, which affirms the fact that hormesis is an endpoint-specific phenomenon, and may not be consistent across all the endpoints [72]. The cells show biphasic dose-response upon ethanol treatment in terms of increase and decrease in MTT activity at low and high doses of ethanol respectively. However, it should also be noted that at 1 % ethanol concentration, though cells retain their viability and showcase high cellular activity, ethanol starts showing an adverse effect on mitochondria and causes cytoskeletal disaggregation. This implies that a dose seemingly stimulatory for one property might be inhibitory for other properties. This observation validates a trade-off in the Darwinian fitness for survival, wherein disruption to cellular homeostasis by a low to moderate stress is counteracted by allocating resources for immediate survival of cells, which may be at the cost of defying other non-essential functionalities [72]. In a larger ecological framework, responses to low-level stress at different organizational levels are also greatly affected by the events of prior exposure to environmental contamination over generations [35,72].

Moreover, the study identifies several structural defects in mitochondria and establishes that cell stiffness is directly related to cell toxicity. It highlights a gradual observable change in cell morphology in a dose-dependent manner starting from actin disorganization and cell membrane instability to a reduction in cell volume and appearance of blebs. These high-dose toxic effects of ethanol seen in cells could be a direct reflection of ethanol toxicity on human health. Similar to the cellular systems, ethanol-induced damage to human health is majorly attributed to the oxidative stress generated due to excessive production of ROS during alcohol metabolism [73]. Moreover, one of the major novel findings of this study is the real-time stress response showcased by cells treated with a sub-toxic level of ethanol; wherein some cells initiate recovery, while others concede to the damage and die. The possibility of the involvement of anti-apoptotic Bcl-2 protein has also been discussed in detail, which paves way for in-depth future studies.

## 5 Conclusion

In conclusion, this study provides a comprehensive understanding of the phenomenon of biphasic-dose response and toxicity of ethanol at the cellular level, with ∼1 % being the concentration of transition. Biphasic-dose response is observed in terms of MTT activity, and toxic effects are evaluated in terms of cell viability and proliferation, morphology, mitochondrial health, and cellular stiffness. A dose-dependent cellular stress response to ethanol toxicity is also a significant original finding of this study. Thus, the observations and inferences drawn from this primary *in vitro* study on altered cellular behavior at low and high doses can not only be translated to animal studies but also should be kept in consideration for *in vitro* toxicological evaluation of medicines and compounds that contain ethanol. Implications of this study could also help explain the variability of response at threshold level across individuals and help in understanding the development of dose tolerance leading to addictive behavior.

## Funding

This research did not receive any specific grant from funding agencies in the public, commercial, or not-for-profit sectors.

## Author contribution statement

Neelakshi Kar: Conceptualization, Investigation, Methodology, Data curation, Writing-original draft; Deepak Gupta: Data curation, Software, Validation, Writing-review, and editing; Jayesh Bellare: Supervisor, Conceptualization, Resources, Validation, Writing-review and editing.

## Conflict of interest

The authors declare no conflict of interest.

## Declaration of Competing Interest

The authors report no declarations of interest.

## Appendix A Supplementary data

Time-lapse imaging of control cells.

Time-lapse imaging of cells exposed to 0.01 % (v/v) ethanol.

Time-lapse imaging of cells exposed to 1 % (v/v) ethanol.

Time-lapse imaging of cells exposed to 3 % (v/v) ethanol.
