# Supplementary material for: Ethanol affects fibroblast behavior differentially at low and high doses: A comprehensive, dose-response evaluation
Source: Toxicol Rep. 2021 May 18;8:1054–66. doi: 10.1016/j.toxrep.2021.05.007 (PMC8296147; doi:10.1016/j.toxrep.2021.05.007)
Supplement: Supplementary file 1 [file mmc1.docx]

**Ethanol affects fibroblast behavior differentially at low and high doses: A comprehensive dose-response evaluation**

Neelakshi Kar^a^, Deepak Gupta^a^, Jayesh Bellare^a,b *^

^a^Department of Chemical Engineering, ^b^Wadhwani Research Centre for Bioengineering,

Indian Institute of Technology Bombay, Powai, Mumbai – 400076, Maharashtra, India

***Corresponding author:**

Prof. Jayesh Bellare

Email: [jb@iitb.ac.in](mailto:jb@iitb.ac.in)

ORCID ID: 0000-0002-6792-8327

Contact number: +91 (22) 2576 7207 (O), +91 (22) 2572 6895 (Fax)

**First author:**

Neelakshi Kar

Email: [neelakshi.kar25@iitb.ac.in](mailto:neelakshi.kar25@iitb.ac.in)

**Second Author:**

Deepak Gupta

Email: [deepakgupta@iitb.ac.in](mailto:deepakgupta@iitb.ac.in)

**Supplementary Information**

**SI 1:** Quantification of cytoskeletal anisotropy was done with the help of the Fibril Tool Plugin of ImageJ. This tool is based on the notion of a nematic tensor which uses raw images from any form of microscopy (here, from fluorescence) to quantify the anisotropy of fiber arrays and their average orientation in cells. The working of this tool can be better explained with the help of a diagram as below.


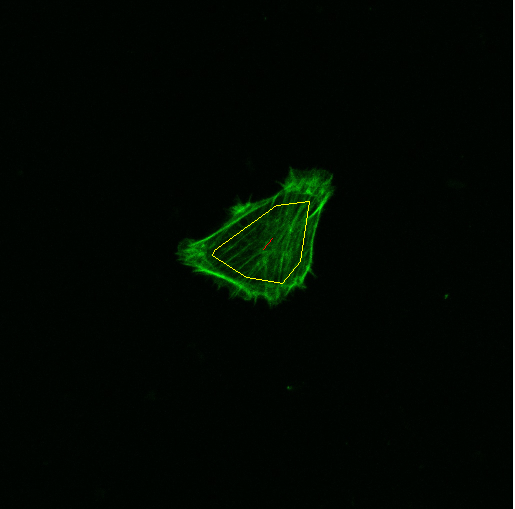


**Figure S1:** Confocal micrograph of fibroblast cell for quantification of anisotropy. To measure the level of anisotropy of actin filaments, the Region of Interest (ROI) is chosen (yellow polygon) in such a way so as to avoid the saturated pixels (shown by white arrows) and overlapping regions of cells (in case of multiple cells in an image). Once the ROI is selected, the FibrilTool plugin generates a line segment (red line) at the center along with an anisotropy value. The length of the red line segment is proportional to anisotropy, the value of which quantifies how parallel the filaments are. This value is independent of the area of ROI selected provided the alignment of fibers is the same.

**SI 2:** This study was designed to systematically understand the effect of ethanol at low as well as high doses. First, the effect of ethanol on MTT Activity was studied over a wide concentration range of 0.005-10 % (v/v) (with 9 concentration points), which yielded a biphasic dose-response curve. Intrigued by this result, we conducted experiments to check if this dose-response is correlated to cell proliferation, taking all concentrations the same as MTT Assay. We then tried to understand if the toxicity observed is related to ROS, thus keeping the concentrations same as the above for DCFDA assay. Based on these experiments, we established that concentrations below 1 % are non-toxic, while above 1 % are toxic. Here the toxic effect of ethanol has been defined in terms of loss of cell viability. So, experiments such as SOD Assay, assays to evaluate mitochondrial health, and time-lapse imaging experiments were done by choosing representative concentrations across the full range, with one concentration from the non-toxic range and, one or two concentration (3 % and 5 %) from toxic range, with 1 % as intermediate concentration. Moreover, cellular morphology (fluorescence and SEM) was studied with the inclusion of some finer grain ethanol concentrations to evaluate the gradual observable change in cellular structures starting with actin disorganization, cell shrinkage, loss of actin, and finally bleb formation signifying cell death. For instance, cellular blebs could only be seen at 7 % concentration, but not much difference in morphology was observed between 0.01 % and 0.05 % concentrations. So, 7 % was considered but 0.05 % was not considered for this study. The same concentrations were kept for the cell stiffness experiment as it is associated with actin disorganization. Nevertheless, there are other practical factors associated, e.g. flow cytometry requires at least 0.5 x 10^6^ cells for analysis, and as we move towards toxic concentration range (> 3 %), the number of cells becomes too low to be analyzed, or the number of live cells could not be calculated for 10 % concentration, because no cell survived.
