# Supplementary material for: Ethanol affects fibroblast behavior differentially at low and high doses: A comprehensive, dose-response evaluation
Source: Toxicol Rep. 2021 May 18;8:1054–66. doi: 10.1016/j.toxrep.2021.05.007 (PMC8296147; doi:10.1016/j.toxrep.2021.05.007)
Supplement: Supplementary file 2 [file mmc2.docx]

**Ethanol affects fibroblast behavior differentially at low and high doses: A comprehensive, dose-response evaluation**

Neelakshi Kar^1^, Deepak Gupta^1^, Jayesh Bellare^1,2 *^

^1^Department of Chemical Engineering, ^2^Wadhwani Research Centre for Bioengineering,

Indian Institute of Technology Bombay, Powai, Mumbai – 400076, Maharashtra, India

**Corresponding author:**

Prof. Jayesh Bellare

Email: [jb@iitb.ac.in](mailto:jb@iitb.ac.in)

ORCID ID: 0000-0002-6792-8327

Contact number: +91 (22) 2576 7207 (O), +91 (22) 2572 6895 (Fax)

**First author:**

Neelakshi Kar

Email: [neelakshi.kar25@iitb.ac.in](mailto:neelakshi.kar25@iitb.ac.in)

**Second Author:**

Deepak Gupta

Email: [deepakgupta@iitb.ac.in](mailto:deepakgupta@iitb.ac.in)

**Supplementary Figure**





Figure S2: MTT Activity of Human Embryonic Kidney (HEK 293) Cells and Hepatocarcinoma (HepG2) cells. The figure indicates that similar to fibroblast cells, an inverted U-shaped curve is also observed for HEK 293 and HepG2 cells upon ethanol exposure.
