# Supplementary material for: Ethanol affects fibroblast behavior differentially at low and high doses: A comprehensive, dose-response evaluation
Source: Toxicol Rep. 2021 May 18;8:1054–66. doi: 10.1016/j.toxrep.2021.05.007 (PMC8296147; doi:10.1016/j.toxrep.2021.05.007)
Supplement: Supplementary file 3 [file mmc3.docx]

**Captions for video files**

Supplementary Material 4 (mmc4): Time-lapse imaging of control cells

Supplementary Material 5 (mmc5): Time-lapse imaging of cells exposed to 0.01 % (v/v) ethanol

Supplementary Material 6 (mmc6): Time-lapse imaging of cells exposed to 1 % (v/v) ethanol

Supplementary Material 7 (mmc7): Time-lapse imaging of cells exposed to 3 % (v/v) ethanol
